# Adaptation of the Educational Motivation Scale Into a Short Form With Multigroup Analysis in a Vocational Training and Baccalaureate Setting

**DOI:** 10.3389/fpsyg.2021.663834

**Published:** 2021-06-04

**Authors:** Jorge Expósito-López, José Javier Romero-Díaz de la Guardia, Eva María Olmedo-Moreno, María Dolores Pistón Rodríguez, Ramón Chacón-Cuberos

**Affiliations:** Department of Methods of Research and Diagnosis in Education, University of Granada, Granada, Spain

**Keywords:** motivation, scale adaptation, vocational training, autodetermination theory, SEM

## Abstract

The aim of the present study was to adapt the educational motivation scale into an abbreviated version (EMS-SF), in addition to analyzing its psychometric properties for use with vocational training (VT) and baccalaureate students using structural equations. A cross-sectional and *ex post facto* study was conducted with a sample of 1,159 students from the autonomous community of Andalusia (Spain). IBM SPSS^®^ and IBM AMOS^®^ software programs were used for data analysis. With regard to the main outcomes, the scale reflected good fit indices in its short form, presenting a more parsimonious and easily understood questionnaire. The questionnaire was reduced from a total of 28–19 items. In the same way, the number of dimensions was reduced from seven to four, facilitating scale understanding and interpretation according to self-determination theory. As a main finding, it was observed that the most relevant items for baccalaureate students pertained to the pleasure derived from discovering things and to overcoming challenges, whereas in VT students, items pertaining to the satisfaction generated from exerting effort, achieving one’s best and being well paid were more relevant. In conclusion, findings urge the need to strengthen intrinsic motivation in VT students with the aim of avoiding demotivation and poor academic performance.

## Introduction

As a field of study, motivation has been approached from various contexts such as the professional, sporting, and educational contexts, among others (Castro-Sánchez et al., 2019; Hayenga and Corpus, 2010). This construct helps to explain the energy that drives a person to perform a task or action, the length of time over which an action is sustained, and whether or not the process leads to satisfaction (Ryan and Deci, 2020). The study of motivation within the academic ambit has been demonstrated by Diseth et al. (2020) and Ryan and Deci (2016) to be essential. Better understanding of the motivational factors acting upon students will help to transform teaching practice in a way that raises levels of motivation and interest. Furthermore, this will enable students to generate a sense of identification with what they are doing, in turn, favoring felt satisfaction, identification with actions and behavioral regulation. All of this will improve academic performance and prevent educational dropout (Núñez et al., 2010; Patall et al., 2018). There is, therefore, an urgent need to develop valid instruments, which enable diagnosis of student motivation.

The present study is grounded in the self-determination theory, developed by Ryan and Deci (2020). This theory states that the motivation felt by an individual at the time of performing a task is established along a “continuum,” which ranges from high to low levels of self-determination. At one extreme of this continuum, intrinsic types of motivation are found (the most self-determined zone), while at the other extreme, demotivation is found (the least self-determined zone). In the middle zone, extrinsic types of motivation can be seen. These are characterized by different levels of internal and external regulation (Gillet et al., 2012; Núñez et al., 2005; Pelletier et al., 2013. Adams et al. (2017) have argued that self-determination theory actually comprises six “mini-theories,” which refer to the multiple aspects that influence an individual’s motivation. Among these, elements pertaining to cognitive evaluation standout such as reward or feedback, goal orientations, locus of causality, and basic psychological needs.

In this way, it is crucial to identify the type and level of motivation in students, in addition to the factors that may influence its development such as autonomy, social support, academic leadership, classroom methods, and the best materials to use (Núñez et al., 2010; Utvær and Haugan, 2016). This will enable the aspects that promote more self-determined motivational types in students to be favored. Indeed, Hayenga and Corpus (2010) and Ryan and Deci (2020) have demonstrated that intrinsically motivated students set goals that are more oriented toward mastery, are more self-efficient, work better as part of a team, and solve problems more effectively. Furthermore, more intrinsically motivated students are better at self-regulating their learning and obtain better academic outcomes. In contrast, extrinsically motivated students develop maladaptive behaviors or stop exerting efforts when external rewards cease (Castro-Sánchez et al., 2019).

Recent studies have shifted focus toward examinations of motivation at school and in postcompulsory secondary educational settings, with the aim of favoring academic performance and the transition toward working life. In this sense, Patall et al. (2018) demonstrated the importance of promoting intrinsic types of motivation in a study conducted with science students. Concretely, students were more autonomous and possessed greater levels of commitment to learning content when they received support from their teachers, were given the chance to ask questions, and were able to choose between various work options according to their preferences. Along these lines, it has also been demonstrated that establishing task-oriented motivational climates favors academic adjustment in adolescents. This creates an ideal predisposition for learning (Castro-Sánchez et al., 2019).

Even at higher stages, such as with vocational training, the importance of motivational development is highlighted. Bacca et al. (2019) demonstrated that when chemistry students completed practical activities, which incorporated augmented reality, various components of motivation were favored such as attention, self-confidence, and satisfaction. Another example is given through the study conducted by Becker et al. (2018). These authors revealed that transforming the educational context so that it was in line with the needs of students led to the development of approach-based goals oriented toward mastery. This reinforced intrinsic motivation and strengthened academic performance.

Thus, the need to obtain effective scales, which permit evaluation of these factors, is highlighted. Currently, there are different scale validation works to assess educational motivation. Many of them focus specifically on elementary educational stages such as primary education or secondary education (Lamb, 2012; Lim and Chapman, 2015), while there are few validations in postcompulsory secondary education (Núñez et al., 2005, 2010). However, these studies present complex factorial structures with a diversity of dimensions, which makes it difficult to identify the type of motivation within the “continuum” of self-determination (Chemolli and Gagné, 2014). For this reason, simple instruments are needed to determine the type of motivation in an effective way, considering the three basic types. Likewise, this validation process will be favored by a decrease in the number of items, making the scale more parsimonious and simpler, as established by Rouquette and Falissard (2011).

Therefore, the present research study pursues the following objectives: (a) To develop an adapted abbreviated version of the educational motivation scale (EMS-SF) in order to facilitate its application within postcompulsory secondary education students; (b) To examine via multigroup analysis which indicators are most relevant to each of the scale dimensions and factors when administered to vocational training and baccalaureate students.

## Materials and Methods

### Design and Participants

The present research work follows a descriptive, exploratory, and cross-sectional design with a sample of 1,159 students [male = 517 (44.6%); female = 642 (55.4%)], undertaking postcompulsory non-university education in the autonomous community of Andalusia, Spain (*M* = 20.59; SD = 6.75). In a specific way, the study sample was composed of 884 VT students (76.3%) and 275 baccalaureate students (23.7%) who were undertaking the aforementioned courses at public centers during the 2019/2020 academic year. It should be noted that the population universe consisted of 291,364 students in high school and VT, which leads us to assume a margin of error of 3% with a confidence level of 95% according to the final sample (*n* = 1,159), being a representative sample. Likewise, the participant selection process was for convenience, applying the following inclusion–exclusion criteria: (a) To be registered for at least 60% of all the course modules required to be completed at the institute; (b) To be regularly attending classes for all evaluated stages; and (c) To not present with any pathology that impedes questionnaire completion. However, and following the indications of Merino-Marban et al. (2015), a certain randomization process can be established when working with natural groups in different educational settings.

### Instruments

The educational motivation scale (EME) validated by Núñez et al. (2005) was used. This original version was developed in the university context and later adapted by Núñez et al. (2010) for use in compulsory secondary education contexts. This scale is composed of a total of 28 items [e.g., *1. Because I need, at least, the title of baccalaureate/certificate of vocational education and training (VET) in order to find a well-paid job*], which are rated along a seven-point Likert type scale where 1 = “Does not correspond at all” and 7 = “Corresponds exactly.” This scale comprises a total of seven dimensions. These pertain to the different levels of educational motivation experienced toward undertaking vocational and baccalaureate training. Namely, demotivation (items 5, 12, 19, and 26), external regulation (items 1, 8, 15, and 22), introjected regulation (items 7, 14, 21, and 28), identified regulation (items 3, 10, 17, and 25), the intrinsic drive for knowledge (items 2, 9, 16, and 23), the intrinsic drive for achievement (items 6, 13, 20, and 27), and the intrinsic drive for stimulating experiences (items 4, 11, 18, and 25). In the original validation study, internal consistency values between α = 0.73 and α = 0.88 were obtained for the scale’s different dimensions. In the present research work, values between α = 0.80 and α = 0.87 were obtained, with these being better than the original values.

### Procedure

The present research adheres to the I + D + i B-SEJ-192-UGR18 research project, funded by the FEDER Fund of the Local Government of Andalusia (Spain).

First, in order to carry out data collection processes inherent to the cross-sectional study, the different educational centers that took part in the present study were notified. For this, the management teams of centers were contacted by an information letter developed by the project’s principal investigator (PI). The letter contained written information about the nature of the study and its objectives, in addition to a commitment to send a report on the study findings at a later date.

Once approval was obtained by the schools’ management, the aforementioned scales were administered during the first 3 months of 2020 in a total of seven educational centers. All young people for whom informed consent was obtained (approval by their legal guardians in case of being minors) participated in the data collection process. Data collection was carried out with the presence of a researcher attached to the project. For each group of students, one of their tutors was also present in order to ensure that scales were correctly filled out and to resolve doubts. This process was completed without any notable incidents.

Finally, it serves to highlight that student anonymity was ensured at all times. Scales were completed anonymously, respecting participants’ rights to confidentiality. It should also be indicated that the present work adhered to the ethical research principles established by the Declaration of Helsinki (1975) and later revendicated in Brazil (2013). The research obtained a positive rating from the Ethical Committee of the University of Granada (reference number: 1678/CEIH/2020).

### Data Analysis

IBM SPSS^®^ version 25.0 software was used to carry out basic descriptive analysis relating to exploratory factor analysis (EFA). Likewise, factor loadings and the rotated matrix were obtained using the software FACTOR Analysis^®^ 9.3.1 (Lorenzo-Seva and Ferrando, 2006). For this, the maximum likelihood (ML) method with varimax rotation was employed for the EFA, using the Cronbach alpha coefficient to determine internal consistency of the scale (95% reliability index). Confirmatory factor analysis (CFA) was performed using the program IBM Amos Graphics^®^. In this case, standardized goodness of fit criteria stipulated by Kock (2014) were employed. In the case of the chi-squared statistic, non-significant *p*-values indicated good model fit. Comparative fit indices (CFI) are considered to be acceptable when the values produced are greater than 0.90 and excellent when values are greater than 0.95. Normalized fit index (NFI) values must be greater than 0.90. Incremental fit indices (IFI) are considered acceptable when the values produced are greater than 0.90 and excellent when values are greater than 0.95. Finally, root mean square error approximation (RMSEA) values are considered excellent when they are lower than 0.05 and acceptable when lower than 0.08. Parameter estimations were performed through the ML method as this is coherent, unbiased and invariant to scale type.

## Results

Table 1 presents average ratings and basic descriptive values pertaining to the 28 items that make up the educational motivation scale for postcompulsory secondary education. Development of this scale was based on the dimensions specified by Davies et al. (2011) and validated by Expósito López et al. (2019). In this sense, different dispersion measures were estimated, considering values of kurtosis and asymmetry with the aim of establishing normality of the responses given to scale items. As a result of this preliminary analysis, items 3 (*Because I think that having undertaken the Baccalaureate/VET course will help me to be better prepared for my chosen profession*), 8 (*In order to get a more prestigious job*), and 10 (*Because it will enable me to access the job market in the field that I most like*) were eliminated. These items were suppressed following the recommendations laid out by Hu and Bentler (1998) and Schmider et al. (2010) as they presented values of kurtosis and asymmetry, which were greater than 2.

**TABLE 1:** Basic descriptive statistics for items of the educational motivation questionnaire administered in vocational training (VT) and baccalaureate students.

Items	*M*	SD	CI	*V*	*A*	*K*
(I.1) Because I need, at least, the title of baccalaureate/certificate of VET in order to find a well-paid job	5.76	1.47	[5.64–5.87]	2.176	–1.368	1.648
(I.2) Because I experience pleasure and satisfaction when I learn new things	5.39	1.47	[5.27–5.50]	2.161	–0.861	0.314
(I.3) Because I believe that having undertaken the baccalaureate/VET will help me to be better prepared for my chosen profession	6.04	1.36	[5.93–6.14]	1.857	–1.828	3.347
(I.4) Because I really like going to class	3.65	1.83	[3.51–3.79]	3.383	0.114	–0.974
(I.5) I honestly don’t know, I think I am wasting my time at school	2.48	1.90	[2.33–2.61]	3.624	1.074	–0.128
(I.6) For the pleasure I experience when I triumph in my studies	4.60	1.83	[4.46–4.73]	3.355	–0.410	–0.824
(I.7) To prove to myself that I am capable of finishing the baccalaureate/VET	4.67	2.15	[4.51–4.83]	4.628	–0.517	–1.114
(I.8) To get a more prestigious job	5.92	1.46	[5.80–6.02]	2.134	–1.594	2.158
(I.9) For the pleasure I feel when I discover new things that I had never seen before	5.12	1.66	[4.99–5.24]	2.754	–0.650	–0.390
(I.10) Because it will enable me to access the job market in the field that I most like	5.98	1.39	[5.87–6.08]	1.944	–1.615	2.366
(I.11) Because for me, school is fun	3.23	1.85	[3.09–3.37]	3.432	0.373	–0.963
(I.12) I used to have good reasons to go to school, but now I ask myself whether it is worth the effort to continue	2.99	2.07	[2.82–3.14]	4.289	0.633	–0.981
(I.13) For the pleasure I feel when I achieve one of my personal objectives	5.19	1.70	[5.06–5.32]	2.920	–0.787	–0.214
(I.14) Because I feel important when I perform tasks well in class	4.43	1.92	[4.28–4.57]	3.707	–0.305	–1.023
(I.15) Because I want to “live well” once I finish my studies	5.87	1.51	[5.75–5.98]	2.292	–1.481	1.693
(I.16) For the pleasure I experience broadening my knowledge about topics that interest me	5.20	1.67	[5.07–5.32]	2.796	–0.796	–0.193
(I.17) Because it will help me to make a better decision in respect to my professional career	5.59	1.55	[5.47–5.70]	2.429	–1.173	0.886
(I.18) For the pleasure I feel when I take part in debates with interesting teachers	4.11	1.91	[3.96–4.25]	3.650	–0.123	–1.074
(I.19) I don’t know why I go to school and, honestly, I don’t care	2.16	1.77	[2.02–2.29]	3.153	1.412	0.785
(I.20) For the satisfaction I feel when I manage to complete difficult academic activities	4.88	1.74	[4.74–5.01]	3.057	–0.568	–0.522
(I.21) To prove to myself that I am an intelligent person	4.52	1.85	[4.38–4.66]	3.422	–0.430	–0.808
(I.22) For the satisfaction I feel when I take on difficult academic activities	5.74	1.53	[5.62–5.85]	2.342	–1.326	1.204
(I.23) Because my studies enable me to keep learning a lot of things which interest me	5.37	1.61	[5.25–5.50]	2.614	–0.906	0.135
(I.24) Because I believe that the education I receive in school will improve my competence at work	5.36	1.68	[5.23–5.48]	2.836	–0.925	0.022
(I.25) Because it drives me to read about topics which interest me	4.69	1.84	[4.55–4.83]	3.406	–0.496	–0.735
(I.26) I don’t know, I don’t understand what I am doing at school	2.23	1.83	[2.08–2.36]	3.378	1.295	0.338
(I.27) Because classes give me personal satisfaction when I strive to get the most out of my studies	4.50	1.81	[4.36–4.63]	3.287	–0.348	–0.809
(I.28) Because I want to prove to myself that I can triumph in my studies	5.29	1.85	[5.15–5.43]	3.454	–0.936	–0.192

*M, mean; SD, standard deviation; CI, confidence intervals; V, variance; A, asymmetry; K, kurtosis.*

**FIGURE 1:**
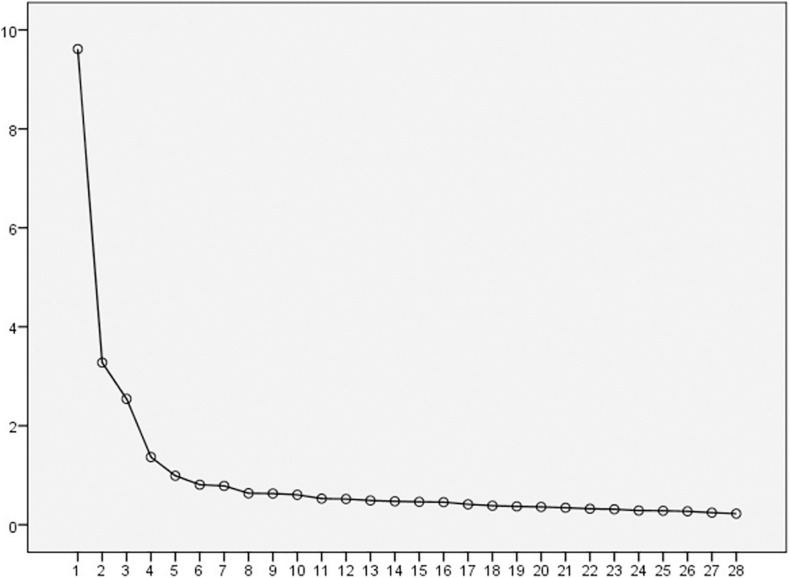
Scree plot.

Next, a scree plot was developed with the aim of verifying the ideal number of factors for the factor solution. From this, the existence of four dimensions or factors can be observed (Figure 1).

Table 2 presents the analysis of the psychometric properties of the scale, considering only the 35 items, or indicators, that were found to have a normal distribution. The program FACTOR Analysis^®^ 9.3.1. was used, employing the ML method (Lorenzo-Seva and Ferrando, 2006) to develop the rotated factor matrix and identify the extent to which the dimensions loaded on the different factors. Adequacy of the correlation matrix was appropriate, as shown through the Bartlett statistic, which had appropriate fit (17,487.39; d*f* = 378; *p* < 0.001), while the Kaiser–Meyer–Olkin (KMO) test value was excellent (KMO = 0.935). Beyond this, the generated four factor solution explained 60.04% of variance in the data, with this being a high percentage. Finally, other indices were employed with the aim of verifying goodness of fit of the rotated solution. Values for the goodness of fit index (GFI) and the adjusted goodness of fit index (AGFI) were excellent, being 0.99 and 0.99, respectively. In the same way, an acceptable value of 0.93 was produced for the comparative fit index (CFI). In this way, it can be established from these data that this factor solution shows excellent fit. Likewise, factor loadings are presented in order from strongest to weakest. Loadings weaker than 0.300 are discarded from this consideration.

**TABLE 2:** Rotated factor matrix and factor loadings.

	Factor 1–Intrinsic motivations	Factor 2–Internal extrinsic motivations	Factor 3–External extrinsic motivations	Factor 4–Demotivation
***I.4***	0.738			
***I.2***	0.737			
***I.9***	0.709			
***I.25***	0.690			
***I.23***	0.676			
***I.16***	0.671			
***I.11***	0.559			
***I.18***	0.551			
***I.28***		0.766		
***I.7***		0.734		
***I.21***		0.728		
***I.20***		0.630		
***I.14***		0.625		
***I.27***		0.604		
***I.6***		0.588		
***I.13***		0.504		
***I.22***			0.818	
***I.1***			0.793	
***I.15***			0.720	
***I.17***			0.497	
***I.24***			0.412	
***I.19***				0.863
***I.26***				0.853
***I.5***				0.808
***I.12***				0.767
***Cronbach alpha***	α = 0.871	α = 0.895	α = 0.859	α = 0.860
***McDonald’s*ω**	ω = 0.869	ω = 0.895	ω = 0.773	ω = 0.859

The final scale was formed by four factors (see Appendix about final scale and dimensions reduced), modifying the seven-factor solution established by Núñez et al. (2010) in the original study. To this end, the following preliminary dimensions were proposed: (1) demotivation, (2) external regulation, (3) introjected regulation, (4) identified regulation, (5) intrinsic drive for knowledge, (6) intrinsic drive for achievement, and (7) intrinsic drive for stimulating experiences. In the new rotated solution, only four dimensions were determined. This simplifies analysis and more effectively aligns it with the self-determination theory (Ryan and Deci, 2017). This factor solution was formed in the following way. Factor 1 was denominated “intrinsic goals” and grouped together the items of *I.2*, *I.4*, *I.9*, *I.11*, *I.16*, *I.18*, *I.23*, and *I.25*. Factor 2 was denominated “internal extrinsic goals” and grouped together the items of *I.6*, *I.7*, *I.13*, *I.14*, *I.20*, *I.21*, *I.27*, and *I.28*. Following this, factor 3 was conceptualized as “external extrinsic goals” and was composed of the items of *I.1*, *I.15*, *I.17*, *I.22*, and *I.24*. Finally, factor 4 was defined as “demotivation” and was composed of the items of *I.5*, *I.12*, *I.19*, and *I.26*. Following this, internal consistency of the overall scale was examined according to the Cronbach alpha coefficient, obtaining high reliability (α = 0.892). Similar values were produced for factor 1 (α = 0.871; ω = 0.869), factor 2 (α = 0.895; ω = 0.895), factor 3 (α = 0.859; ω = 0.773), and factor 4 (α = 0.860; ω = 0.859) (Table 2).

Next, CFA was performed with the aim of comparing outcomes with the scale consistency outcomes specified using EFA. To this end, a structural equation model (SEM) was developed, which was formed by the different determined factors, and the items comprised by each of them. The model was composed by a total of four latent variables, which represented the obtained dimensions, alongside a total of 25 observable variables, which related to the items included in the final scale. First, model fit indices are reported for this scale. A significant chi-squared value was obtained (*X*^2^ = 2,400.47; d*f* = 269; *p* < 0.001]. Nonetheless, it serves to highlight that this test presents a high degree of sensitivity to sample size. For this reason, it was decided to employ other fit indices. In this regard, the IFI, NFI, and CFI were also used. All of these indices revealed cautiously acceptable values (IFI = 0.858; NFI = 0.842; CFI = 0.857). On the other hand, the RMSEA value produced was acceptable (0.079).

It serves to indicate that these values are not considered excellent according to guidelines established by Kock (2014); Marsh et al. (2020), and Arbuckle (2011) establishes how the specification of the model helps the researcher to fit it to the data. For this, one of the parameters to consider are the modification indices (MI), which are associated with a reduction in the chi-square value (MI > 3.84 suggests a statistically significant reduction in *X*^2^ when estimating the coefficient). For this reason, modification indices of the regression weights were reviewed, eliminating those with values greater than 20. The aim of this was to improve fit indices and obtain a more synthetic scale for the four factors. Indeed, Adams et al. (2017) previously warned of the challenges that present themselves at the time of attempting to differentiate between the different factors that make up motivation in relation to the self-determination theory. This obliged the aforementioned action to be taken. The eliminated indicators pertained to items 11, 13, 17, 18, 21, and 24. In this way, acceptable fit indices were obtained (*X*^2^ = 823.59; d*f* = 146; *p* < 0.001; IFI = 0.938; NFI = 0.926; CFI = 0.938; RMSEA = 0.064). Likewise, internal consistency was again determined for the scale following consideration of this new distribution of indices. The overall scale produced a value of α = 0.830 (ω = 0.828), while partial values for the factors were α = 0.872 and ω = 0.872 (factor 1), α = 0.866 and ω = 0.866 (factor 2), α = 0.801 and ω = 0.801 (factor 3), and α = 0.804 and ω = 0.859 (factor 4).

**FIGURE 2:**
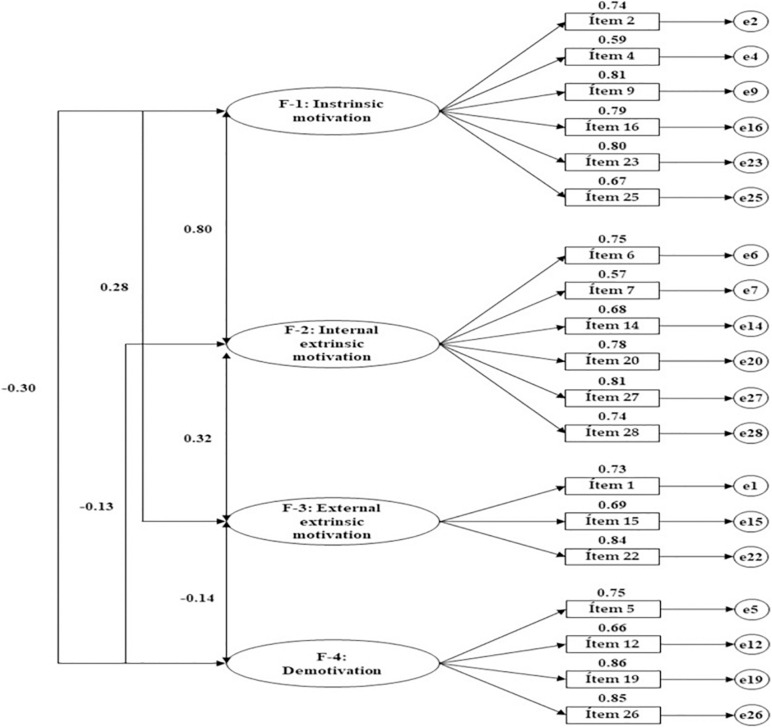
Structural model for the confirmatory analysis.

Table 3 and Figure 2 present the standardized regression weights produced in the structural model pertaining to the associations between the four factors and their indicators. These weights relate to the overall sample. The statistics reveal significant positive associations (*p* < 0.05) for all indicators with their relevant factor. In relation to “intrinsic motivation” (factor 1), regression weights indicated that the indicator to exert the greatest influence was item 9 (*For the pleasure I experience when I discover new things I had never seen before*) (*b* = 0.812; *p* < 0.005). In contrast, the least influential item was item 4 (*Because I really like going to class*) (*b* = 0.593; *p* < 0.005). In the case of “internal extrinsic motivation” (factor 2), the greatest regression weight was produced for item 27 (*Because classes give me personal satisfaction when I strive to get the most out of my studies*) (*b* = 0.815; *p* < 0.005), while the smallest coefficient was produced for item 7 (*To prove to myself that I am capable of finishing the baccalaureate/VT*) (*b* = 0.575; *p* < 0.005). Finally, “external extrinsic motivation” (factor 3) found item I.22 (*In order to achieve a better salary later on*) to be of most dimensional relevance (*b* = 0.840; *p* < 0.005), while item 19 (*I don’t know why I go to school and, honestly, I don’t care*) was most important to factor 4 “demotivation” (*b* = 0.862; *p* < 0.005).

**TABLE 3:** Standardized regression weights for scale items from the developed model.

Association between items and their relevant factor			RW				SRW
			
			Estimation	SE	CR	*p*	Estimation
I.2	←	F-1	1.000	−	−	***	0.737
I.4	←	F-1	1.007	0.052	19.454	***	0.593
I.9	←	F-1	1.244	0.046	27.033	***	0.812
I.16	←	F-1	1.223	0.046	26.354	***	0.793
I.23	←	F-1	1.194	0.045	26.622	***	0.801
I.25	←	F-1	1.143	0.052	22.129	***	0.671
I.6	←	F-2	1.000	−	−	***	0.746
I.7	←	F-2	0.905	0.048	18.869	***	0.575
I.14	←	F-2	0.957	0.043	22.518	***	0.679
I.20	←	F-2	1.003	0.038	26.216	***	0.784
I.27	←	F-2	1.081	0.040	27.315	***	0.815
I.28	←	F-2	1.005	0.041	24.631	***	0.739
I.1	←	F-3	1.000	−	−	***	0.731
I.15	←	F-3	0.974	0.048	20.453	***	0.694
I.22	←	F-3	1.193	0.056	21.291	***	0.840
I.5	←	F-4	1.000	−	−	***	0.752
I.19	←	F-4	1.069	0.038	28.389	***	0.862
I.26	←	F-4	1.091	0.039	28.122	***	0.850
I.12	←	F-4	0.958	0.044	21.797	***	0.663
F-2	↔	F-3	0.467	0.056	8.277	***	0.318
F-4	↔	F-2	–0.254	0.067	–3.805	***	–0.130
F-4	↔	F-1	–0.465	0.056	–8.258	***	–0.300
F-4	↔	F-3	–0.213	0.055	–3.891	***	–0.138
F-1	↔	F-3	0.322	0.044	7.368	***	0.276
F-1	↔	F-2	1.190	0.075	15.830	***	0.805

RW, regression weight; SRW, standardized regression weight; SE, standard error; CR, critical ratio; F-1, factor 1–intrinsic motivations; F-2, factor 2–internal extrinsic motivations; F-3, factor 3–external extrinsic motivations; F-4, factor 4–demotivation. ***Statistically significant differences at the level of *p* < 0.005.

Finally, the structural model developed from the CFA analyzed the associations generated between dimensions from the empirical data. In this case, the strongest relationship was seen between factor 1 and factor 2 (*b* = 0.805; *p* < 0.005). This was followed by a moderate–weak association between factor 2 and factor 3 (*b* = 0.318; *p* < 0.005). Both of these associations were positive. In contrast, when factor 4 was linked with demotivation, negative regression weights were produced in relation to all of the remaining dimensions: Factor 1 (*b* = −0.300; *p* < 0.005), factor 2 (*b* = −0.130; *p* < 0.005), and factor 3 (*b* = −0.138; *p* < 0.005).

Following this, with the aim of contributing data that are more in line with the contextual reality of the two analyzed groups (students undertaking vocational training and baccalaureate; Tables 4, 5), multigroup analysis was performed considering both educational levels. In this case, indices of model fit were acceptable. Concretely, specified values were as follows: *X*^2^ = 1,038.62; d*f* = 292; *p* < 0.001; IFI = 0.932; NFI = 0.908; CFI = 0.931; RMSEA = 0.047.

**TABLE 4:** Standardized regression weights for vocational training students.

Association between items and their relevant factor			RW				SRW
			
			Estimation	SE	CR	P	Estimation
I.2	←	F-1	1.000	−	−	***	0.726
I.4	←	F-1	1.047	0.074	11.024	***	0.560
I.9	←	F-1	0.987	0.063	15.738	***	0.794
I.16	←	F-1	1.222	0.055	22.394	***	0.787
I.23	←	F-1	1.231	0.055	22.198	***	0.783
I.25	←	F-1	1.176	0.053	22.091	***	0.660
I.6	←	F-2	1.000	−	−	***	0.733
I.7	←	F-2	1.123	0.060	18.579	***	0.586
I.14	←	F-2	0.982	0.050	19.700	***	0.694
I.20	←	F-2	0.981	0.045	21.675	***	0.761
I.27	←	F-2	1.084	0.047	23.035	***	0.809
I.28	←	F-2	1.035	0.048	21.540	***	0.757
I.1	←	F-3	1.000	−	−	***	0.711
I.15	←	F-3	0.991	0.056	17.561	***	0.692
I.22	←	F-3	1.222	0.067	18.334	***	0.849
I.5	←	F-4	1.000	−	−	***	0.752
I.19	←	F-4	1.080	0.044	24.797	***	0.868
I.26	←	F-4	1.076	0.044	24.265	***	0.841
I.12	←	F-4	0.936	0.049	18.989	***	0.662
F-2	↔	F-3	0.539	0.066	8.144	***	0.373
F-4	↔	F-2	–0.126	0.075	–1.684	0.092	–0.065
F-4	↔	F-1	–0.403	0.062	–6.455	***	–0.266
F-4	↔	F-3	–0.207	0.064	–3.249	**	–0.132
F-1	↔	F-3	0.406	0.051	7.918	***	0.360
F-1	↔	F-2	1.082	0.081	13.353	***	0.775

*Note 1: RW, regression weight; SRW, standardised regression weight; SE, standard error; CR, critical ratio.*

*Note 2: ^∗∗^, statistically significant differences at the level of *p* < 0.01; ^∗∗∗^, statistically significant differences at the level of *p* < 0.005.*

*Note 3: F-1, factor 1 – intrinsic motivations; F-2, factor 2 – internal extrinsic motivations; F-3, factor 3 – external extrinsic motivations; F-4, factor 4 – demotivation.*

**TABLE 5:** Standardized regression weights for baccalaureate students.

Association between items and their relevant factor			RW				SRW
			
			Estimation	SE	CR	*p*	Estimation
I.2	←	F-1	1.000	−	−	***	0.743
I.4	←	F-1	1.047	0.098	10.685	***	0.652
I.9	←	F-1	1.308	0.094	13.977	***	0.836
I.16	←	F-1	1.260	0.095	13.231	***	0.795
I.23	←	F-1	1.299	0.093	13.966	***	0.835
I.25	←	F-1	1.190	0.109	10.916	***	0.665
I.6	←	F-2	1.000	−	−	***	0.772
I.7	←	F-2	0.823	0.092	8.910	***	0.541
I.14	←	F-2	0.878	0.084	10.390	***	0.622
I.20	←	F-2	1.082	0.073	14.860	***	0.846
I.27	←	F-2	1.088	0.075	14.577	***	0.832
I.28	←	F-2	0.967	0.081	11.905	***	0.701
I.1	←	F-3	1.000	−	−	***	0.807
I.15	←	F-3	0.884	0.086	10.252	***	0.689
I.22	←	F-3	1.068	0.099	10.824	***	0.792
I.5	←	F-4	1.000	−	−	***	0.761
I.19	←	F-4	1.028	0.073	14.025	***	0.843
I.26	←	F-4	1.140	0.079	14.516	***	0.882
I.12	←	F-4	1.031	0.095	10.854	***	0.665
F-2	↔	F-3	0.263	0.108	2.427	*	0.177
F-4	↔	F-2	–0.675	0.146	–4.624	***	–0.349
F-4	↔	F-1	–0.674	0.121	–5.577	***	–0.448
F-4	↔	F-3	–0.256	0.106	–2.423	*	–0.177
F-1	↔	F-3	0.166	0.083	1.999	*	0.144
F-1	↔	F-2	1.375	0.166	8.261	***	0.890

*RW, regression weight; SRW, standardized regression weight; SE, standard error; CR, critical ratio; NF-1, factor 1–intrinsic motivations; F-2, factor 2–internal extrinsic motivations; F-3, factor 3–external extrinsic motivations; F-4, factor 4–demotivation.*

*^∗^Statistically significant differences at the level of *p* < 0.05.*

*^∗∗∗^Statistically significant differences at the level of *p* < 0.005.*

**FIGURE 3:**
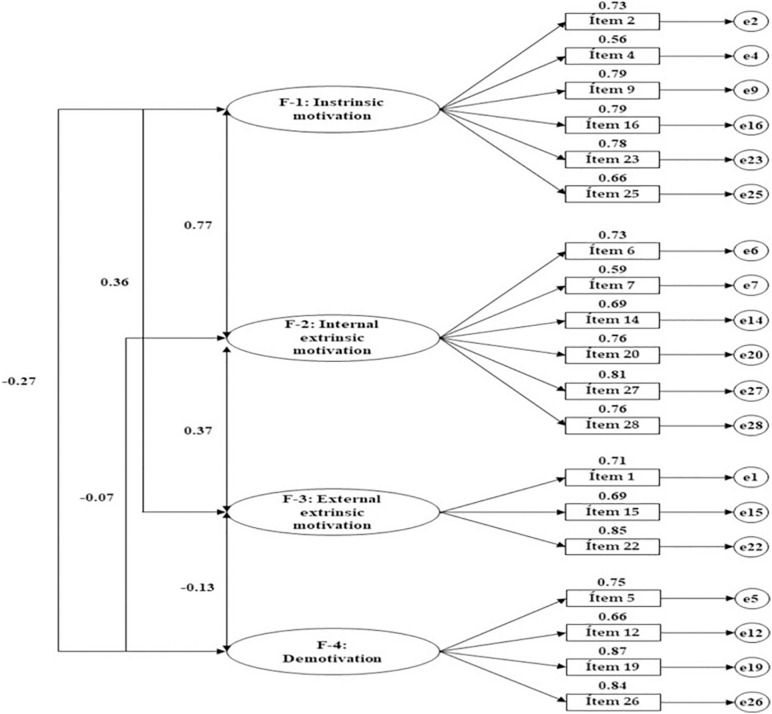
Structural model for the vocational training students.

Table 3 and Figure 2 present the standardized regression weights produced by the structural model for associations between the four factors and their indicators in the present sample of vocational training students. On the other hand, Table 4 and Figure 3 presents outcomes pertaining to students who were undertaking baccalaureate courses. Perusal of the statistics reveals significant positive values (*p* < 0.05) for the associations produced between all indicators and their relevant factors in both groups. With regard to “intrinsic motivation” (factor 1), regression weights indicate that the indicator to exert the greatest influence is item 9 (*For the pleasure I feel when I discover new things that I had never seen before*). This was the most relevant indicator for this factor in both groups, although a stronger weight is found within baccalaureate students (*b* = 0.794; *p* < 0.005 vs. *b* = 0.836; *p* < 0.005). The least influential item is item 4 (*Because I really like going to class*). Once again, a stronger regression weight is seen in the second group (*b* = 0.586; *p* < 0.005 vs. *b* = 0.652; *p* < 0.005).

Reviewing factor 2 “internal extrinsic motivation,” the strongest regression weight is seen in relation to item 27 (*Because classes give me personal satisfaction when I strive to get the most out of my studies*) in the case of vocational training student (*b* = 0.809; *p* < 0.005). In baccalaureate students, item 20 (*For the satisfaction I feel when I manage to complete difficult academic activities*) produced the highest value (*b* = 0.846; *p* < 0.005). In both groups, the weakest regression weight was produced for item 7 (*To prove to myself that I am capable of finishing a baccalaureate/VET*) (*b* = 0.586; *p* < 0.005 vs. *b* = 0.541; *p* < 0.005).

For factor 3 “external intrinsic motivation,” item I.22 (*To earn a better salary later on*) was the item that demonstrated greatest influence within the group of vocational training students (*b* = 0.840; *p* < 0.005), with item 1 (*Because I need, at least, the title of baccalaureate/certificate of VET to find a well-paid job*) being the most relevant within the baccalaureate group (*b* = 0.801; *p* < 0.005). In both groups, item 15 (*Because I want to “live well” once I finish my studies*) was the least influential item for this dimension (*b* = 0.692; *p* < 0.005 vs. *b* = 0.689; *p* < 0.005). Finally, with regard to factor 4 “demotivation,” item 19 (*I don’t know why I go to school and, honestly, I don’t care*) was the most relevant within VT students (*b* = 0.868; *p* < 0.005), while item 26 (*I don’t know, I don’t understand what I am doing at school*) presented the greatest regression weight within baccalaureate students (*b* = 0.882; *p* < 0.005).

Finally, the structural model developed from the CFA analyses the associations relating to the dimensions generated from the empirical data. In this case, the strongest association was produced between factor 1 and factor 2, with the coefficient being greatest among baccalaureate students (*b* = 0.775; *p* < 0.005 vs. *b* = 0.890; *p* < 0.005). Likewise, an inverse association is seen between factor 1 and factor 4, with this association being weaker among VT students (*b* = 0.266; *p* < 0.005 vs. *b* = -0.448; *p* < 0.005). An association is also seen between factor 1 and factor 3, which is more prominent within VT students than baccalaureate students (*b* = 0.360; *p* < 0.005 vs. *b* = 0.144; *p* < 0.05). Finally, a significant association is indicated between factor 2 and factor 4 among baccalaureate students (*b* = -0.349; *p* < 0.005), while this association was not significant among VT students (*p* = 0.092).

## Discussion

An exploratory study was conducted of an adapted version of the educational motivation scale in the context of postcompulsory secondary education. The aim of this was to develop an abbreviated version of this scale and examine the influence of its various indicators as a function of the educational stage of respondents: vocational training and baccalaureate. The present research work is of huge importance due to existing needs for an abbreviated and parsimonious scale. It follows a similar approach to that taken in work conducted by Gagné et al. (2015), Núñez et al. (2005), Núñez et al. (2010), Pelletier et al. (2013), and Utvær and Haugan (2016).

The process of analysis employed to examine the psychometric properties of the abbreviated form of the scale followed recommendations laid out by Lorenzo-Seva and Ferrando (2006), Marsh et al. (2020), and Schmitt (2011). First, basic descriptive statistics pertaining to questionnaire items were analyzed according to their mean scores, asymmetry, and kurtosis with the aim of identifying data dispersion. In this regard, a total of three variables were eliminated (items 3, 8, and 10) as they presented values that are equal to or greater than 2. Elimination of all questions whose response trends were not in line with the general responses of the sample made the questionnaire more synthetic.

Next, EFA was performed. A scree plot was developed with the aim of identifying the most appropriate factor solution, proposing a model that was based on four factors. The original scale was grouped into seven factors or dimensions, defining the diverse levels specified by Adams et al. (2017) and based on the self-determination theory: demotivation, three types of extrinsic motivation (external, introjected, and identified regulation), and three types of intrinsic motivation (drive for knowledge, drive for achievement, and drive to obtain stimulating experiences). Given that the self-determination theory defines motivation along a “continuum” (Ryan and Deci, 2016), it seems logical that analysis based on seven motivational types may be confusing, or even generate factor loading issues given the proximity of all dimensions.

The final factor structure was made up of a total of four factors or dimensions, comprised of a total of 25 items. No single indicator was eliminated for having a factor loading lower than 0.400, while excellent fit indices were obtained for KMO, GFI, and CFI values. Within this new factor distribution, motivational types were simplified. The structures of factor 4 (demotivation) and factor 1 (intrinsic motivations), associated with stimulating experiences, were retained. However, in the new scale structure, two factors were proposed for extrinsic motivational types: Factor 2 “internal extrinsic motivations” and factor 3 “external extrinsic motivations.” The existence of these factors was supported by the factor loadings of the items. This may be explained by the fact that the original description of intrinsic goals for achievement and knowledge classified these items as internally originating extrinsic goals; however, the items associated with external regulation are linked with extrinsic goals that are external in nature (Pelletier et al., 2013; Utvær and Haugan, 2016).

Following EFA, CFA was conducted. In this way, an adjusted structural model was obtained for the factors and items. Furthermore, the modification indices of the regression weights were reviewed, and those with values greater than 20 were eliminated. The purpose of this was to improve fit indices and obtain a more synthetic scale for the four factors. Indeed, Ryan and Deci (2016) previously outlined existing difficulties at the time of differentiating between the different components that make up motivational levels in the self-determination theory. This justifies the need to develop a shortened version of the scale. In this case, removed indicators pertained to items 11, 13, 17, 18, 21, and 24.

By briefly analyzing the associations between the different items and factors in the final scale, it can be observed that factor 1 “intrinsic motivation” was mainly determined by the pleasure derived from describing things and the interest for learning. As established by Castro-Sánchez et al. (2019), these are two basic elements that strengthen self-determination for an action. In relation to factor 2 “internal extrinsic motivation,” the strongest regression weight was associated with obtaining personal satisfaction from getting the most out of activities and bettering oneself. These relate to less self-determined factors, although they are internally regulated. In contrast, factor 3 “external extrinsic motivation” was mainly associated with externally regulated elements such as getting a good job or a high wage (Ryan and Deci, 2020). Likewise, the relationships between factors reveal negative associations between all motivational types and factor 4 “demotivation.” The most prominent relationship was seen between internally regulated intrinsic and extrinsic motivations. This may largely be due to the focus on personal identification, which is inherent in these motivational types (Diseth et al., 2020).

**FIGURE 4:**
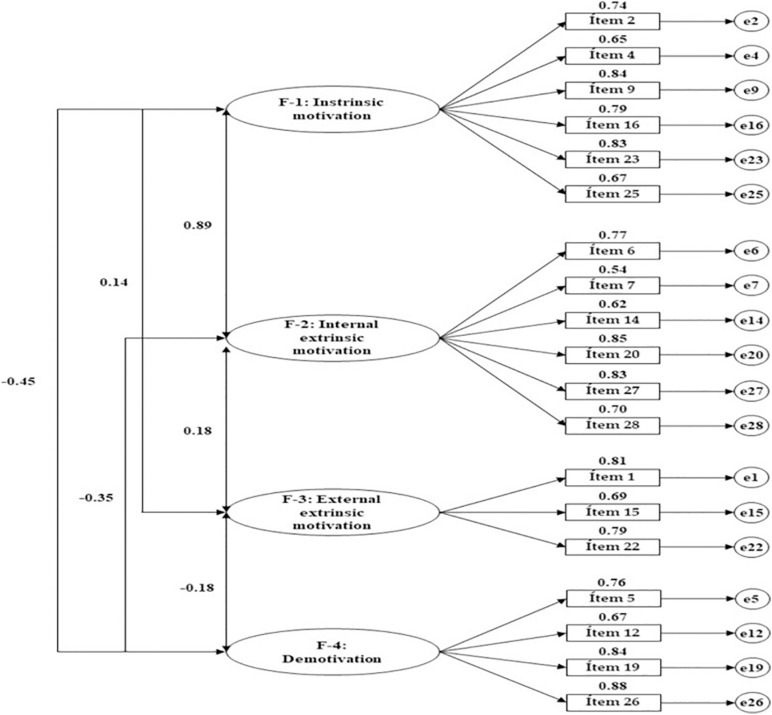
Structural model for the baccalaureate students.

It is relevant to give a special mention to the outcomes of the multigroup analysis according to the two analyzed educational stages, VT and baccalaureate. Generally speaking, baccalaureate students produced greater regression weights in regard to the indicators of intrinsic motivation (Figure 4). This could denote a better fit in relation to these motivational types. Such outcomes could be explained by the curricular itinerary that characterizes this stage, with this material paving the way toward university qualifications. This assumes a more vocational focus through VT, permitting more direct access to the job market (Hayenga and Corpus, 2010; Gillet et al., 2012). This same trend can be seen with regard to internal extrinsic motivations. Indeed, baccalaureate students tend to report greater satisfaction from overcoming difficult activities, suggesting, in this way, that they have greater self-determination (Rego-Agraso and Rial-Sánchez, 2017).

Following review of the dimensions linked to less self-determined zones, it was seen that VT students produced greater regression weights in relation to motivations for achieving a good salary, with this being associated with external extrinsic motivations. A similar situation was seen in baccalaureate students; however, these students placed greater focus on the type of work that needs to be performed in order to achieve economic rewards. This superseded integral rewards, in this way being associated with a more external type of regulation (Núñez et al., 2010; Utvær and Haugan, 2016). Along these lines, it seems intuitive that VT students report better fitting values for indicators of demotivation. This is true, especially, among those who show no interest in the motives for undertaking this course. This situation often leads to the abandonment of behaviors that are exclusively sustained by extrinsic motivations as, once the motivational element ceases to exist, frustration, disinterest, and dropout prevail (Pelletier et al., 2013; Ryan and Deci, 2016).

In conclusion, it is essential to indicate the main limitations of the present study. The sample used could be overly contextualized due to the fact that participants were only included from the autonomous community of Andalusia (Spain). Furthermore, distribution was non-homogenous with regard to representation of VT and baccalaureate students, despite a large sample being used. As a further limitation, we can also point to the complexity of the studied construct–motivation and self-determination theory. Thus, the definition of scale dimensions was a complicated task. Nonetheless, it serves to indicate the high relevance of the conducted study. Indeed, the present study simplified a complex scale for its administration with children, reducing it from 28 items and seven dimensions to a final total of 19 items and four dimensions. This was done without neglecting any element of the self-determination theory and produced acceptable fit indices.

The method chosen to develop the CFA should also be noted as a limitation, since the ML method is used. This method is adequate because it is consistent, unbiased, and invariant to the type of scale. However, there are other methods such as the diagonal weighted least squares (DWLS) and weighted least squares–mean (WLSM) that provide more precise factorial loads and more robust statistics. Even so, Li (2016) specifies how the ML method is valid for these research works, specially, for samples with more than 1,000 subjects, such as Arbuckle (2011) establishes how the specification of the model helps the researcher to fit it to the data. For this, one of the parameters to consider is the MI, which is associated with a reduction in the chi-square value (MI > 3.84 suggests a statistically significant reduction in X2 when estimating the coefficient). As future perspectives, it is intended to use a different statistical software package in order to apply these methods to perform the CFA, such as LISREL 8.80. Moreover, there are obvious applications of conducting a stratified study according to educational stages, while also broadening analysis to the national context. This will enable the psychometric properties of the scale to be examined in a broader sample. Regarding this, it would also be essential to apply the reduced scale in a different sample in order to check the discriminant validity of the scale effectively. Furthermore, other scales should be used to further verify its convergent and content validity.

## Conclusion

The educational motivation scale (EMS) showed good fit and reliability indices in its short form (EMS-SF), proposing a more parsimonious and easy-to-understand questionnaire for postcompulsory secondary education students. The final scale was reduced from a total of 28 items to 19, being cut from a total of seven dimensions to four. This facilitates scale understanding and interpretation according to the self-determination theory: intrinsic motivation, internal extrinsic motivation, external extrinsic motivation, and demotivation. Fit indices pertaining to EFA and CFA were excellent, and internal consistency of the dimensions was better than that of the original version. Furthermore, hugely relevant findings emerged with regard to comparisons between the estimation of constructs in VT and baccalaureate students. The pleasure of discovering new things was more relevant for intrinsic motivation in baccalaureate students. Likewise, the satisfaction of overcoming challenges was more important for internal extrinsic motivation within this group, while the satisfaction acquired from pushing oneself to achieve the best was more relevant with VT students. The economic factor was more relevant for externally regulated extrinsic motivations in VT students. Furthermore, a closer relationship was observed between internally regulated intrinsic and extrinsic motivations in baccalaureate students, with these also harvesting a greater extent of demotivation. This premises have important educational implications for both stages and point to the types of motivation that should be targeted within baccalaureate and VT students. From this, the need to reinforce internally regulated intrinsic motivations in VT students should be emphasized, with the aim of avoiding course dropout or demotivation, instead favoring self-determined elements that ensure success and academic achievement.

## Data Availability Statement

The raw data supporting the conclusions of this article will be made available by the authors, without undue reservation.

## Author Contributions

JE-L and RC-C conceived the hypothesis of this study. All authors contributed to data collection. JE-L, EO-M, and RC-C analyzed the data. All authors contributed to data interpretation of the statistical analysis. All authors read and approved the final manuscript.

## Conflict of Interest

The authors declare that the research was conducted in the absence of any commercial or financial relationships that could be construed as a potential conflict of interest.

## Appendix

**APPENDIX:** Final Scale And Dimensions Reduced.

I-O	I-SF	Descriptor	Factor
1	**1**	Because I need, at least, the title of baccalaureate/certificate of VET in order to find a well-paid job	**3-EEM**
2	**2**	Because I experience pleasure and satisfaction when I learn new things	**1-IM**
4	**3**	Because I really like going to class	**1-IM**
5	**4**	I honestly don’t know, I think I am wasting my time at school	**4-D**
6	**5**	For the pleasure I experience when I triumph in my studies	**2-IEM**
7	**6**	To demonstrate to myself that I am capable of finishing the baccalaureate/VET	**2-IEM**
9	**7**	For the pleasure I feel when I discover new things that I had never seen before	**1-IM**
12	**8**	I used to have good reasons to go to school, but now I ask myself whether it is worth the effort to continue	**4-D**
14	**9**	Because I feel important when I perform tasks well in class	**2-IEM**
15	**10**	Because I want to “live well” once I finish my studies	**3-EEM**
16	**11**	For the pleasure I experience broadening my knowledge about topics that interest me	**1-IM**
19	**12**	I don’t know why I go to school and, honestly, I don’t care	**4-D**
20	**13**	For the satisfaction I feel when I manage to complete difficult academic activities	**2-IEM**
22	**14**	For the satisfaction I feel when I take on difficult academic activities	**3-EEM**
23	**15**	Because my studies enable me to keep learning a lot of things which interest me	**1-IM**
25	**16**	Because it drives me to read about topics which interest me	**1-IM**
26	**17**	I don’t know, I don’t understand what I do at school	**4-D**
27	**18**	Because classes give me personal satisfaction when I strive to get the most out of my studies	**2-IEM**
28	**19**	Because I want to prove to myself that I can triumph in my studies	**2-IEM**

*I-O, original item; I-SF, short-form item; 1-IM, intrinsic motivation; 2-IEM, internal extrinsic motivation; 3-EEM, external extrinsic motivation; 4-D, demotivation.*
